# Cross‐material memory prediction: Influence of preoperative visual memory on postoperative verbal memory change after left anterior temporal lobe resection in people with epilepsy

**DOI:** 10.1002/epi.70426

**Published:** 2026-08-10

**Authors:** Lea Marie Reisch, Anne Hagemann, Johanna Lena Hopf, Florian J. Muecke, Friedrich G. Woermann, Christian G. Bien, Philip Grewe

**Affiliations:** ^1^ Department of Epileptology, Krankenhaus Mara, Bethel Epilepsy Center, Medical School OWL Bielefeld University Bielefeld Germany; ^2^ Society for Epilepsy Research Bielefeld Germany; ^3^ Department of Neuropsychology and Rehabilitation Psychology, Donders Institute for Brain, Cognition, and Behavior Radboud University Nijmegen the Netherlands; ^4^ Clinical Neuropsychology and Epilepsy Research, Medical School OWL Bielefeld University Bielefeld Germany; ^5^ Medical Psychology, Medical School OWL Bielefeld University Bielefeld Germany

**Keywords:** epilepsy surgery, neuropsychology, prediction, temporal lobe epilepsy, verbal memory, visual memory

## Abstract

**Objective:**

Left anterior temporal lobe resection (TLR) in people with epilepsy is associated with a high risk of postoperative verbal memory decline. Whereas substantial evidence supports the role of preoperative verbal memory in predicting postoperative verbal memory change, the potential contribution of preoperative visual memory has been rarely investigated.

**Methods:**

We retrospectively analyzed data from 105 people with left TLR due to mesial temporal sclerosis and typical language dominance. Multiple linear and logistic regression analyses were conducted to predict the extent and risk of significant postoperative verbal memory decline based on preoperative verbal and visual memory performance, as well as clinical variables (age at surgery, age at onset, and preoperative focal to bilateral tonic–clonic seizures). In exploratory analyses, we examined the influence of dichotomized preoperative verbal and visual memory performance (impaired vs. unimpaired) on the extent and frequency of postoperative verbal memory decline, as well as the potential influence of general intellectual ability.

**Results:**

Regression analyses showed that higher preoperative verbal memory performance was associated with more pronounced and more frequent postoperative verbal memory decline, whereas higher preoperative visual memory performance was associated with less severe and less frequent verbal memory decline. Clinical variables did not improve model prediction. Analyses with dichotomized preoperative verbal and visual memory performance confirmed the results of the regression analyses. General intellectual ability had an additional influence on postoperative verbal memory change, without changing the predictive value of preoperative verbal or visual memory performance.

**Significance:**

These findings demonstrate a cross‐material relationship between preoperative visual memory and postoperative verbal memory change following left TLR. Whereas preoperative verbal memory performance likely represents functional adequacy, preoperative visual memory appears to represent functional reserve. Therefore, our findings reconcile these two concepts of postoperative verbal memory change. Additionally, the influence of general intellectual ability suggests relevance of more general cognitive reserve.

**Key points**

- We investigated cross‐material effects of preoperative visual memory on postoperative verbal memory change after left temporal lobe resection.
- Intact preoperative verbal memory was associated with more pronounced and more frequent postoperative verbal memory decline.
- Intact preoperative visual memory was associated with less pronounced and less frequent postoperative verbal memory decline.
- Additionally, general intellectual ability was associated with less pronounced and less frequent verbal memory decline.
- Findings reconcile the concepts of functional adequacy and reserve, which both seem to influence postoperative verbal memory change.

## 1 INTRODUCTION

Epilepsy surgery is an effective treatment for medically refractory temporal lobe epilepsy,^1^ resulting in seizure freedom in approximately two thirds of cases.^2^ Unfortunately, particularly following temporal lobe resection (TLR) in the left hemisphere, people with epilepsy are at high risk of postoperative verbal memory decline.^3^ However, there is substantial interindividual variability in the extent and direction of postoperative memory change, with some people showing a marked decline whereas others demonstrate stable or even improved verbal memory following left TLR, depending on various preoperative predictors.^4^

Numerous studies investigated influences of various clinical variables on postoperative verbal memory change after left TLR. Besides resection of the hippocampus^5^, ^7^ and larger extent of the resection,^8^ negative effects of older age at surgery^5^, ^9^, ^10^ and epilepsy onset,^4^, ^11^ and the presence of preoperative focal to bilateral tonic–clonic seizures^6^ have been demonstrated. Most consistently, it has been demonstrated that postoperative verbal memory change depends strongly on preoperative verbal memory performance, with higher preoperative scores predicting poorer memory outcomes.^4^, ^5^, ^7^, ^9^, ^13^ Imaging studies further showed that greater verbal memory decline following left TLR is predicted by left‐lateralized activation in the medial temporal lobe (MTL) during word‐encoding tasks,^14^, ^15^ and by left‐lateralized white matter network asymmetry in the MTL.^16^ Similarly, Wada test scores indicating stronger left hemisphere memory dominance were associated with greater verbal memory decline.^17^, ^18^

Only few studies have included neuropsychological measures other than verbal memory when predicting postoperative verbal memory change following left TLR. The reason for this may be the still strong impact of the model of material‐specific memory lateralization in the temporal lobes in the field of cognitive outcomes in temporal lobe surgery.^19^ However, it is widely accepted by now that this model represents an oversimplification, because there is no absolute lateralization of verbal and nonverbal memory, and memory tests rarely measure verbal or nonverbal memory performance exclusively.^19^, ^20^ In this context, a cross‐material influence of preoperative visual memory on postoperative verbal memory change may be particularly relevant. St‐Laurent et al.^21^ reported that higher preoperative visual memory performance predicted less postoperative decline in verbal memory after left TLR, suggesting a direction of effect contrary to the effect of preoperative verbal memory on postoperative verbal memory change. By contrast, Dupont et al.^22^ found no contribution of preoperative visual memory performance to postoperative verbal memory change. Notably, sample sizes in these studies were relatively small (both *N* < 30), particularly given the use of multivariate statistical approaches. Functional magnetic resonance imaging (fMRI) studies have also yielded contradictory results. Some studies reported cross‐material associations between lateralization of preoperative MTL activation during visual memory tasks and postoperative verbal memory decline,^22^, ^23^ others did not.^24^, ^25^ Therefore, the influence of preoperative visual memory performance on postoperative verbal memory change after left TLR remains inconclusive. Besides memory functions, some studies included preoperative general intellectual abilities (i.e., intelligence quotient [IQ]) in their prediction of postoperative verbal memory decline,^5^, ^6^, ^9^, ^22^ two of which showed that lower IQ predicted verbal memory decline following left^5^ and right^9^ TLR.

The aim of the present study was to investigate the cross‐material contribution of preoperative visual memory performance to the prediction of postoperative verbal memory change in a large and homogeneous sample of people with typical language dominance who underwent left TLR. We hypothesized that in addition to better preoperative verbal memory performance predicting greater verbal memory decline, better preoperative visual memory performance would be associated with less verbal memory decline. Furthermore, we aimed to develop a multivariate prediction model incorporating preoperative verbal and visual memory measures as well as clinical variables available at presurgical evaluation, and explored the influence of general intellectual ability on postoperative verbal memory change.

## 2 MATERIALS AND METHODS

### 2.1 Participants

The sample was drawn from a clinical database of all people who underwent surgery for temporal lobe epilepsy at the Department of Epileptology (Krankenhaus Mara, Bethel Epilepsy Center), Bielefeld University, Germany, between 2003 and 2020 and who were at least 14 years old at the time of surgery (Figure 1). To ensure a homogeneous sample, only cases with left‐sided anteromedial TLR due to mesial temporal sclerosis (MTS) were included. Left‐sided anteromedial TLR involved the removal of the temporal pole and temporolateral cortex caudal from the temporal pole (approximately 2 cm of the superior temporal gyrus, 3 cm of the middle temporal gyrus, and 4 cm of the inferior temporal gyrus). The hippocampus, amygdala, and parahippocampal gyrus (including the entorhinal cortex) were removed, posteriorly projecting onto a point just anterior of the aqueduct. With two neurosurgeons covering the resections of the sample, standard extent of the anteromedial TLR has not been changed over the inclusion period. Additional inclusion criteria were typical (i.e., left‐hemispheric) language dominance, as determined by fMRI,^26^ and availability of both pre‐ and postoperative verbal and visual memory data (see Section 2.2). People with prior brain surgery or insufficient German language proficiency were excluded. The final sample comprised 105 individuals. Sociodemographic and clinical characteristics of the sample are presented in Table 1. The study protocol was approved by the ethics committee of the Ärztekammer Westfalen‐Lippe, Münster (no. 024‐229‐f‐S), which granted a waiver of informed consent in accordance with North Rhine‐Westphalian law on health data protection.

**FIGURE 1:**
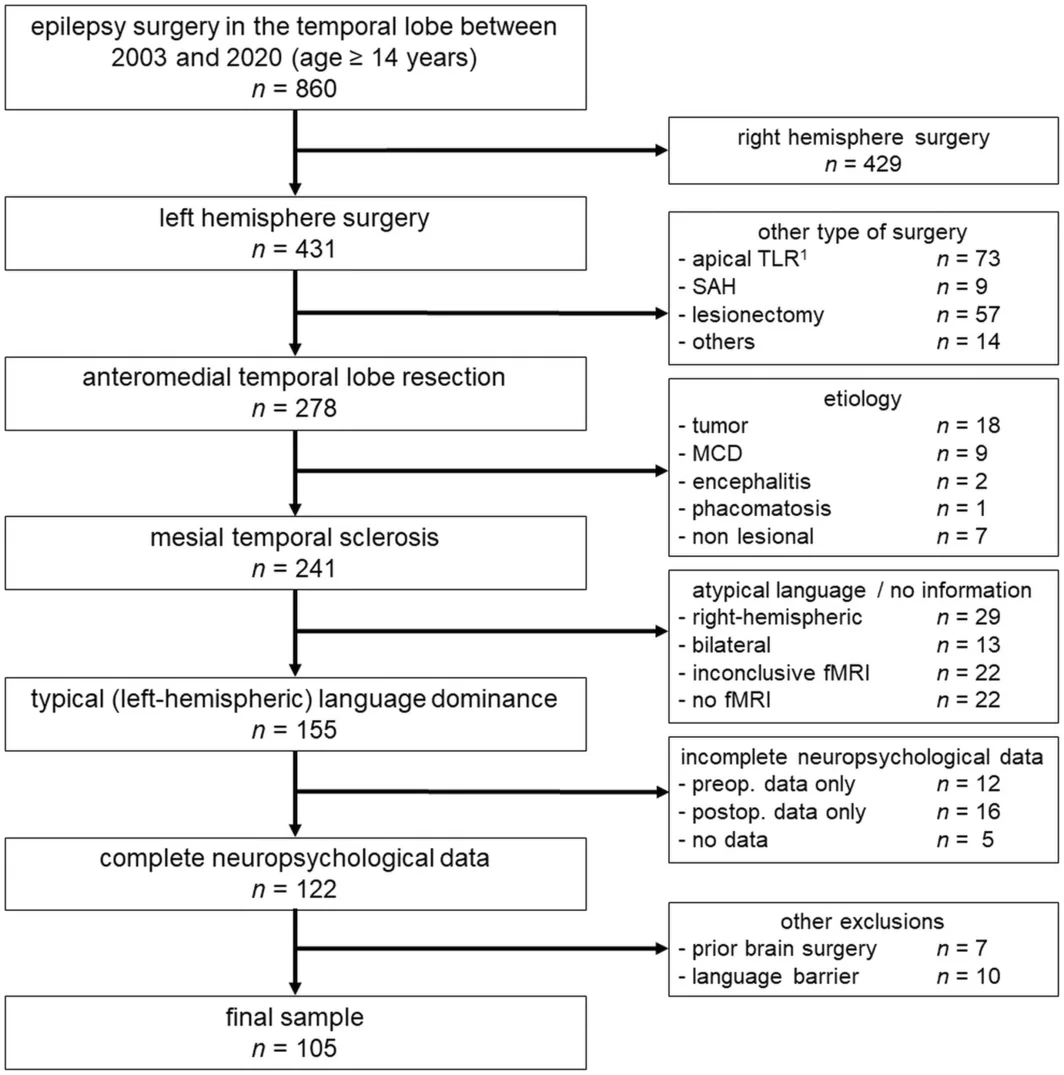
Sample selection and exclusion process. fMRI, functional magnetic resonance imaging; MCD, malformation of cortical development; SAH, selective amygdalohippocampectomy; TLR, temporal lobe resection. ^1^Hippocampus‐sparing, tailored resection of the apex of the temporal lobe.

**TABLE 1:** Participants' demographic and clinical data (*N* = 105).

Sex, female/male	56/49
Handedness, right/left/ambidextrous	97/5/3
Age at epilepsy onset, years	13.56 (11.98)
History of focal to bilateral tonic–clonic seizures, no/yes	36/69
Age at surgery, years	37.22 (13.44)
Epilepsy duration, years	23.65 (14.55)
Seizure outcome (Engel class), I/II/III/IV	85/11/5/4
Time between preoperative NP assessment and surgery, months	2.90 (4.11)
Time between surgery and postoperative NP assessment, months	5.99 (1.43)
Time between pre‐ and postoperative NP assessments, months	9.36 (4.28)

*Note*: Data are presented as *n* or mean (SD). Epilepsy duration was determined by the difference between age at epilepsy onset and surgery.

Abbreviation: NP, neuropsychology.

### 2.2 Neuropsychological measures

People with epilepsy underwent a comprehensive neuropsychological assessment as part of the standard presurgical evaluation and the postsurgical follow‐up 6 months after surgery. Verbal memory was assessed using the Verbal Learning and Memory Test,^27^ a German adaptation of the Rey Auditory Verbal Learning Test.^28^ In this list‐learning paradigm, 15 words are recalled after each of five consecutive learning trials. Following a single presentation and recall of a distractor list of 15 unrelated words, immediate free recall and, after 30 min, delayed free recall of the previously learned list are performed, followed by a forced‐choice recognition test. For the analyses, we used the number of words recalled in the delayed free recall, because this score reflects both learning and memory performance. Visual memory was assessed with the Diagnosticum für Cerebralschädigung.^29^ In this test, nine geometric figures consisting of five straight lines are presented over five consecutive learning trials. After each trial, as many figures as possible must be reproduced using five wooden sticks. The total number of correctly reproduced figures across all five learning trials represents visual memory performance. Both the delayed recall score of the Verbal Learning and Memory Test and the total learning score of the Diagnosticum für Cerebralschädigung have previously been used as valid measures to detect material‐specific memory deficits in people with temporal lobe epilepsy.^30^ For comparability, preoperative verbal and visual memory test scores were transformed into *z*‐scores based on the means and SDs of the respective normative samples.^27^, ^29^ Details on the tests' normative samples are reported in the supplements. *Z*‐scores of <−1 were classified as “impaired,” as suggested by the manual of the Verbal Learning and Memory Test,^27^ whereas *z*‐scores of ≥−1 were classified as “intact.”

In the postoperative neuropsychological assessment, parallel versions of both memory tests were administered. The extent of postoperative verbal memory change was calculated by subtracting preoperative from postoperative raw scores, because changes in raw scores are less susceptible to floor effects. Negative values indicate postoperative decline, whereas positive values indicate postoperative gain in the number of words recalled after the 30‐min delay. Significant verbal memory changes were determined using a reliable change index (RCI) at a 90% confidence interval, based on the test manual.^27^ A loss of three or more words in delayed recall indicates a deterioration, whereas a gain of three or more words indicates an improvement in verbal memory.

As part of an exploratory analysis, general intellectual ability was estimated based on one measure of logical reasoning and one measure of vocabulary, with tests varying over the period of data collection. In most cases, logical reasoning was assessed with the Leistungsprüfsystem subtest 4^31^ and vocabulary with the Mehrfachwahl‐Wortschatz‐Intelligenztest.^32^ In fewer cases, the matrix reasoning and similarities subtest of the German version of the Wechsler Adult Intelligence Scale‐IV^33^ were used. All test scores were transformed into *z*‐scores based on the normative data of the respective test, and mean *z*‐scores for logical reasoning and vocabulary were used as “IQ estimate.”

### 2.3 Statistical analysis

Data were analyzed using IBM SPSS Statistics 25. Differences between preoperative verbal and visual memory performance (*z*‐scores) were examined using a paired *t*‐test. The relationship between *z*‐scores of preoperative verbal and visual memory performance was assessed with Pearson correlation, and the association between the presence of preoperative verbal and visual memory impairments (*z*‐scores < −1) was analyzed using a chi‐squared test. Across the entire sample, the extent of postoperative verbal memory change was assessed using a paired *t*‐test, and the frequency of significant postoperative verbal memory changes was examined according to the RCI. To predict the individual extent of postoperative verbal memory change, a hierarchical multiple regression model was constructed. Preoperative verbal memory performance (*z*‐score) was entered in the first block, followed by preoperative visual memory performance (*z*‐score) in the second block. Based on previous literature, age at surgery,^5^, ^9^, ^10^ age at epilepsy onset,^4^, ^11^ and the presence of preoperative focal to bilateral tonic–clonic seizures^6^ were included in a third block. The same predictors were entered blockwise into a logistic regression analysis to predict the risk of significant postoperative verbal memory decline according to the RCI. In an exploratory analysis, we reanalyzed our data with dichotomized independent variables based on the presence or absence of preoperative verbal and/or visual memory impairments (*z*‐scores of <−1). The influence of preoperative verbal and/or visual memory impairments on the extent of postoperative verbal memory change was investigated using a two‐factorial analysis of variance (ANOVA; verbal memory impairment × visual memory impairment). Effects on the frequency of significant postoperative verbal memory decline according to the RCI were analyzed using a three‐way hierarchical log‐linear analysis (preoperative verbal memory impairment × preoperative visual memory impairment × postoperative verbal memory decline).

The influence of preoperative general intellectual ability on postoperative verbal memory was assessed in an exploratory analysis only including cases for which at least one test on logical reasoning and one test on vocabulary (see Section 2.2) were available. First, correlations of IQ estimate with preoperative verbal and visual memory performance as well as with postoperative verbal memory change were examined. Second, IQ estimate was included as predictor in the multiple linear and logistic regression, entered in an additional block after inclusion of preoperative verbal and visual memory performance, and before inclusion of the clinical variables.

## 3 RESULTS

### 3.1 Preoperative memory performance

There was no significant difference between preoperative verbal (mean = −1.03, SD = 1.40) and visual (mean = −.97, SD = 1.28) memory performance, *t*_104_ = −.39, *p* = .694, *d* = −.04 (Figure 2A). Verbal memory impairments were observed in 42.86% and visual memory impairments in 46.67% of cases (Figure 2B). Even though *z*‐scores of verbal and visual memory were correlated (*r* = .31, *p* = .002), there was no significant association between the presence of verbal and visual memory impairments, *χ* ^2^_1_ = 1.41, *p* = .323, *φ* = .12.

**FIGURE 2:**
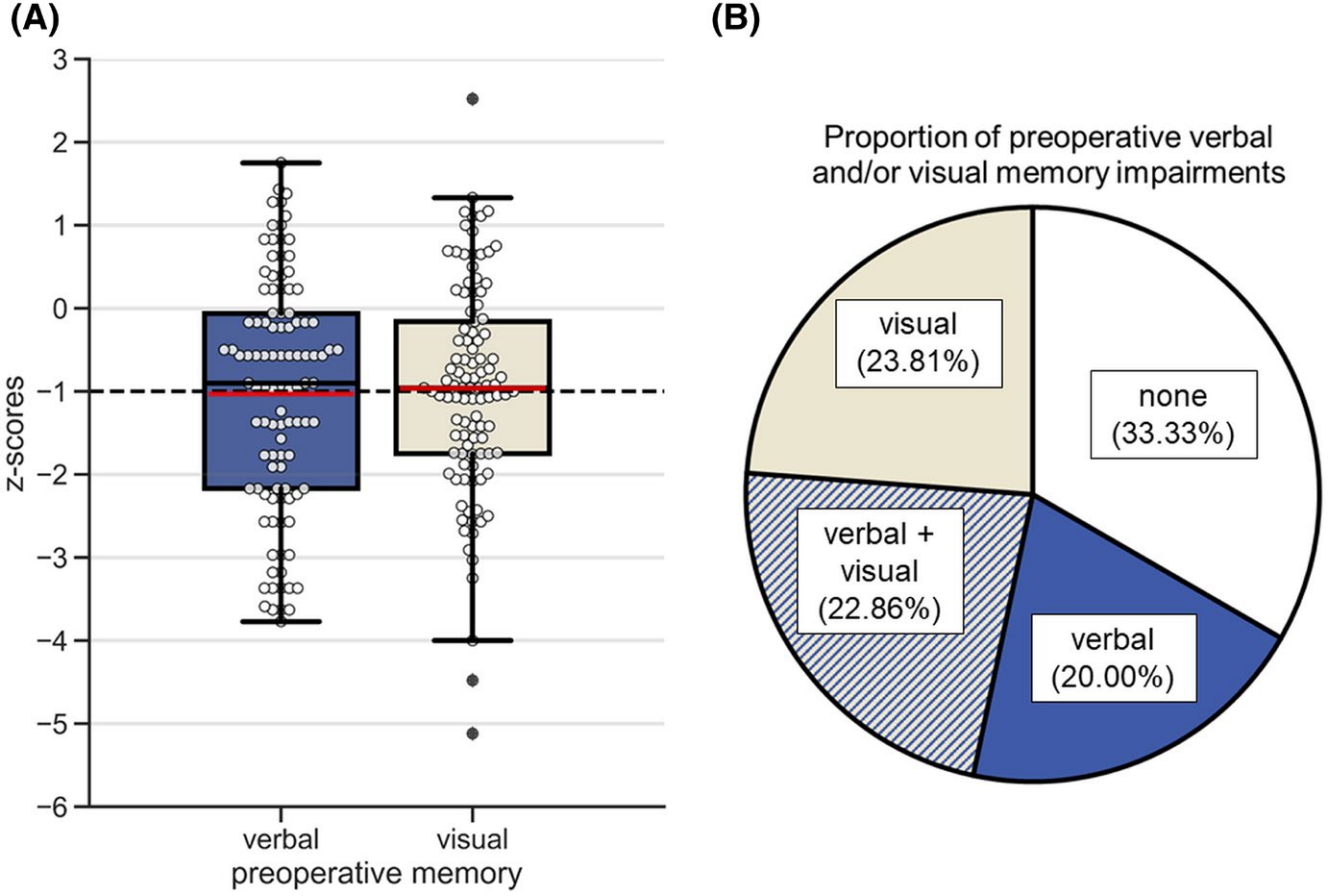
Preoperative verbal and visual memory performance. (A) Preoperative memory performance is expressed as *z*‐scores. Red lines indicate the mean *z*‐score for the respective memory test. Dashed line indicates cutoff for memory impairments. (B) Proportion of preoperative memory impairments (*z*‐scores < −1).

### 3.2 Postoperative verbal memory changes

#### 3.2.1 Extent and frequency of verbal memory change

There was a significant postoperative decline in the number of words recalled in the delayed free recall (mean = −1.57, SD = 2.80), *t*_104_ = −5.75, *p* < .001, *d* = −.56. According to the RCI, 33.33% of the cases showed a significant verbal memory decline, whereas memory performance remained stable in 59.05% and improved in 7.62%.

#### 3.2.2 Prediction of extent of postoperative verbal memory change

There was no indication of multicollinearity among predictor variables (all variance inflation factors < 1.22). Model summaries and regression coefficients for the multiple linear regression predicting the extent of postoperative verbal memory change are presented in Table 2. Preoperative verbal memory performance significantly predicted postoperative verbal memory change. Adding preoperative visual memory performance in the second block significantly improved model fit, whereas inclusion of clinical variables in the third block did not further improve prediction.

**TABLE 2:** Results of hierarchical multiple linear regression to predict extent of postoperative verbal memory change.

	*B*	SE *B*	*β*	*p*	*R* ^2^	*p* (*∆R* ^2^)
Block 1					.13	<.001
(constant)	−2.32	.32		<.001		
Preoperative verbal memory	−.73	.18	−.37	<.001		
Block 2					.23	.001
(constant)	−1.85	.33		<.001		
Preoperative verbal memory	−.92	.18	−.46	<.001		
Preoperative visual memory	.69	.20	.32	.001		
Block 3					.25	.339
(constant)	−1.23	.75		.106		
Preoperative verbal memory	−.95	.18	−.48	<.001		
Preoperative visual memory	.70	.20	.32	.001		
Age at surgery	−.01	.02	−.04	.684		
Age at epilepsy onset	−.03	.02	−.14	.124		
Preoperative focal to bilateral tonic–clonic seizures	.17	.53	.03	.747		

*Note*: *B*, unstandardized coefficient, *β*, standardized coefficient, *R* ^2^, determination coefficient, ∆*R* ^2^, change in *R* ^2^.

#### 3.2.3 Prediction of significant postoperative verbal memory change

Model summaries and regression coefficients for the logistic regression are presented in Table 3. Postoperative verbal memory decline according to the RCI was significantly predicted by preoperative verbal memory in the first block. The addition of preoperative visual memory in the second block significantly improved the model. Again, adding clinical variables in the third block did not improve the model. The model including only preoperative verbal and visual memory performance explained 13.65% of the variability in postoperative verbal memory change (Nagelkerke *R* ^2^). Using a cutoff value of .25, consistent with previous studies,^12^, ^17^ the model correctly classified 60.00% of the cases, with a sensitivity of 91.43% and a specificity of 44.29%.

**TABLE 3:** Results of logistic regression to predict significant postoperative verbal memory decline according to the reliable change index.

	*B*	SE *B*	Odds ratio	*p*	*χ* ^*2*^	*p* (*∆χ* ^*2*^)
Block 1					5.77	.016
(constant)	−.36	.25		.151		
Preoperative verbal memory	.37	.16	1.45	.021		
Block 2					10.86	.024
(constant)	−.65	.29		.026		
Preoperative verbal memory	.50	.18	1.65	.005		
Preoperative visual memory	−.40	.19	.67	.029		
Block 3					14.06	.361
(constant)	−1.44	.71		.042		
Preoperative verbal memory	.53	.19	1.70	.004		
Preoperative visual memory	−.40	.19	.67	.036		
Age at surgery	.01	.02	1.01	.736		
Age at epilepsy onset	.02	.02	1.02	.213		
Preoperative focal to bilateral tonic–clonic seizures	.36	.50	1.43	.474		

*Note*: B, unstandardized coefficient.

*χ* ^2^, Model *χ* ^2^; ∆*χ* ^2^, change in *χ* ^2^.

### 3.3 Exploratory analyses

#### 3.3.1 Verbal memory change depending on preoperative memory impairments

Figure 3 illustrates postoperative verbal memory change as a function of preoperative verbal and/or visual memory impairments. The ANOVA revealed a main effect of preoperative verbal memory impairment on the extent of postoperative verbal memory change, *F*_1, 101_ = 17.55, *p* < .001, *η*_*p*_^2^ = .15. People with intact preoperative verbal memory (mean = −2.42, SD = 2.70) showed more pronounced verbal memory decline than those with preoperative verbal memory impairments (mean = −.44, SD = 2.55). A main effect of preoperative visual memory impairment was also observed, *F*_1, 101_ = 8.59, *p* = .004, *η*_*p*_^2^ = .08. People with intact preoperative visual memory (mean = −1.02, SD = 2.93) exhibited less postoperative verbal memory decline than those with preoperative visual memory impairments (mean = −2.20, SD = 2.52). The interaction between preoperative verbal and visual memory impairments was not significant, *F*_1, 101_ = .68, *p* = .412, *η*_*p*_^2^ = .01.

**FIGURE 3:**
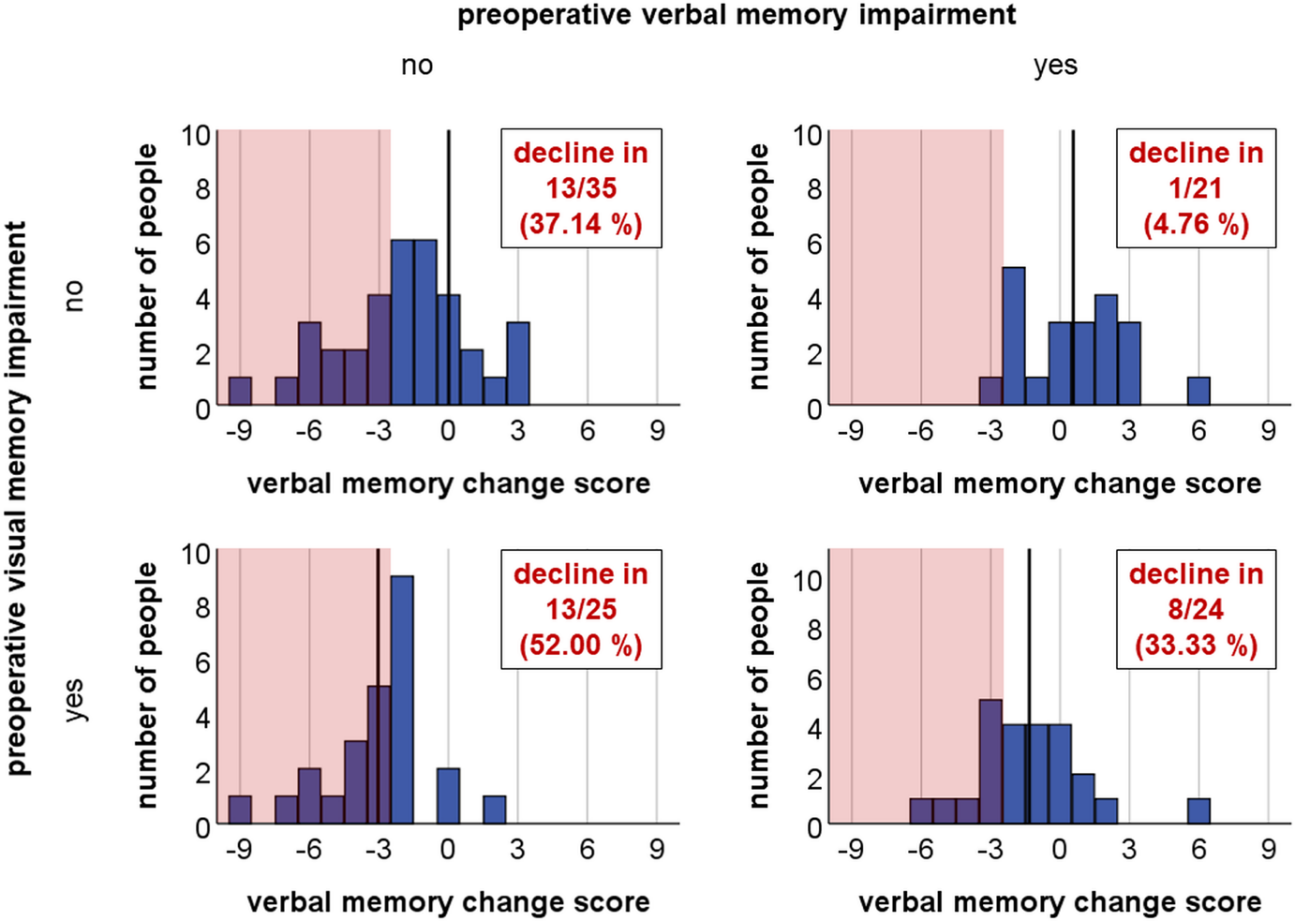
Postoperative verbal memory change (change scores: post − pre) as a function of preoperative verbal and/or visual memory impairments. Bold black lines indicate subgroup means of verbal memory change. Shaded red areas indicate significant verbal memory decline (≤−3 words) according to the reliable change index at a 90% confidence interval.

Detailed results of the hierarchical log‐linear analysis are presented in the supplements (Table S1). Log‐linear analysis yielded a final model retaining only the two‐way interaction between preoperative verbal memory impairment and postoperative verbal memory change as well as the corresponding main effects. However, with a likelihood ratio of *χ* ^2^_4_ = 9.63, *p* = .047, there was a significant deviation between expected and observed frequencies. Therefore, we adopted the last model fitting our data with a likelihood ratio of *χ* ^2^_2_ = 5.40, *p* = .067. This model included the two‐way interaction both between preoperative verbal memory impairment and postoperative verbal memory change, and between preoperative visual memory impairment and postoperative verbal memory change, as well as corresponding main effects. However, the interaction between preoperative visual memory impairment and postoperative verbal memory in this model just failed to reach significance (*p* = .055). Parameter estimates for the adopted model are presented in the supplements (Table S2). For people with intact preoperative verbal memory, the odds of postoperative verbal memory decline were 3.06 times higher than for those with preoperative verbal memory impairments. Conversely, the odds of postoperative verbal memory decline were 2.25 times higher in people with preoperative visual memory impairments than in those with intact preoperative visual memory.

#### 3.3.2 Influence of general intellectual ability on verbal memory change

Estimates of IQ were available for 80 people. Exploratory analyses indicated that IQ estimate was correlated with neither preoperative verbal (*r* = .16, *p* = .167) nor visual (*r* = .09, *p* = .444) memory performance, but higher IQ estimates were associated with less postoperative verbal memory decline (*r* = .23, *p* = .042). Results of the multiple linear and logistic regression analyses including IQ estimate as additional predictor are presented in the supplements (Tables S3 and S4). In the multiple linear regression, inclusion of IQ estimate significantly improved the model compared to the model including preoperative verbal and visual memory as the only predictors (*p* = .003), while verbal and visual memory remained significant predictors. In the logistic regression, inclusion of IQ estimate similarly improved the model (*p* = .002), but the effect of preoperative visual memory was not significant in this model (*p* = .124), whereas verbal memory remained a significant predictor of postoperative verbal memory decline (*p* = .001). However, as compared to the respective model in the original full sample of 105 cases, preoperative visual memory already showed a smaller effect in this analysis in a reduced sample (i.e., 80 cases with available IQ estimates) before including IQ estimate. Therefore, the lower effect of visual memory appeared to be caused by the lower statistical power rather than by inclusion of IQ estimate.

## 4 DISCUSSION

The present findings demonstrate that both the extent and frequency of postoperative verbal memory decline following left TLR depend not only on preoperative verbal memory but also on preoperative visual memory performance. In line with previous findings, better preoperative verbal memory performance predicted more pronounced and more frequent verbal memory decline. Conversely, higher preoperative visual memory performance was associated with less severe and less frequent verbal memory decline. Clinical variables (i.e., age at surgery, age at epilepsy onset, and the presence of preoperative focal to bilateral tonic–clonic seizures) did not contribute to the prediction. Exploratory reanalysis of the data with dichotomized predictors based on the presence or absence of preoperative verbal and/or visual memory impairments confirmed the results of the regression analyses. Higher general intellectual ability had an additional positive effect on postoperative verbal memory change.

The finding of cross‐material memory prediction is consistent with the results of St‐Laurent et al.,^21^ while addressing several limitations of that study. First, our sample comprised 105 people, compared with 28 people with left TLR in the previous study. Second, St‐Laurent et al.^21^ included different surgical procedures (i.e., anterior TLR and selective amygdalohippocampectomy), which may have differential effects on postoperative verbal memory outcomes.^8^, ^34^ By contrast, we selected a homogeneous sample of people who underwent anteromedial TLR only. Third, St‐Laurent et al.^21^ applied principal component analysis to derive latent verbal and visual memory components from multiple neuropsychological test scores. Although composite scores may provide more stable estimates of underlying cognitive constructs, prediction models based on individual test scores may be more applicable in a clinical context, because the approximate risk of postoperative verbal memory decline can be derived from neuropsychological test scores without complex calculations. Finally, we not only examined continuous change scores but also replicated our findings using a dichotomous outcome variable (i.e., significant memory decline according to the RCI), thereby placing greater emphasis on meaningful change at the individual level.

The cross‐material relationship between preoperative visual memory performance and postoperative verbal memory change supports a more unified and less material‐specific concept of memory. However, the direction of effect seems to be material‐specific. Whereas high preoperative verbal memory performance represents a risk factor for postoperative verbal memory decline, high preoperative visual memory conversely seems to be a protective factor. A possible interpretation of this protective effect is that visual memory performance mainly reflects contralateral (i.e., right) temporal lobe memory capacity, which may support verbal memory following surgery according to the functional reserve hypothesis.^35^ Supporting this interpretation, good right‐hemispheric memory performance for pictorial material in the Wada test is associated with less verbal memory decline after left TLR.^36^ Similarly, fMRI studies reported cross‐material relationships between preoperative activation in the MTL^22^ or hippocampus^23^ during visual memory tasks and postoperative verbal memory change, with left‐lateralized activation predicting greater verbal memory decline. At first sight, these fMRI findings mainly seem to support the functional adequacy hypothesis, assuming that postoperative memory decline is determined by the preoperative memory capacity of the MTL structures that are resected.^35^ However, these studies examined laterality indices aggregating left and right activation into a single difference score. Therefore, the same findings conversely imply that right‐lateralized visual memory activation is associated with less verbal memory decline, thus also supporting the functional reserve hypothesis. Even though commonly seen as opposing theories, functional adequacy and functional reserve may rather be complementary mechanisms influencing postoperative verbal memory change at the same time. This is also supported by a resting‐state fMRI study showing that both the functional integration of the temporal lobe structures to be resected in a broader memory network (i.e., functional adequacy), and the functional connectivity within a degenerate memory network spanning distributed regions of both hemispheres (i.e., functional reserve) determine postoperative verbal memory change.^37^ The two concepts appear particularly reconcilable, when assuming only partial material‐specific memory lateralization in the temporal lobes,^20^ and that performance in “visual memory tests” may partly result from verbal encoding strategies and vice versa.^19^ Within the traditional framework, preoperative verbal memory performance in the present study may be best interpreted as representing functional adequacy of the left temporal lobe. This accounts well for the postoperative verbal memory decline after left TLR. At the same time, preoperative visual memory performance may be explained as indicating functional reserve capacities, thus counteracting verbal memory decline. The cross‐material nature of this relationship may be explained in three different ways. First, performance in the visual memory test may truly reflect visual memory capacity of the contralateral temporal lobe that can facilitate compensatory mnemonic strategies for verbal memory tasks, such as visual imagery.^38^ Second, performance in the visual memory test may partly reflect “atypical” verbal memory capacity of the right temporal lobe, because people commonly verbalize the figures in the visual memory test used in the present study. Third, performance in the visual memory test might reflect a more general, supramodal memory competence supported by a degenerate memory network.^37^ An overview over the potential mechanisms explaining the present results is given in Figure 4. Of note, these different aspects are not mutually exclusive, and it is not possible to determine their relative contribution to the present findings. Studies including verbal and visual memory fMRI, separately analyzing left and right temporal lobe activation and systematic recording of the use of memory strategies, might further help to elucidate the exact underlying mechanisms.

**FIGURE 4:**
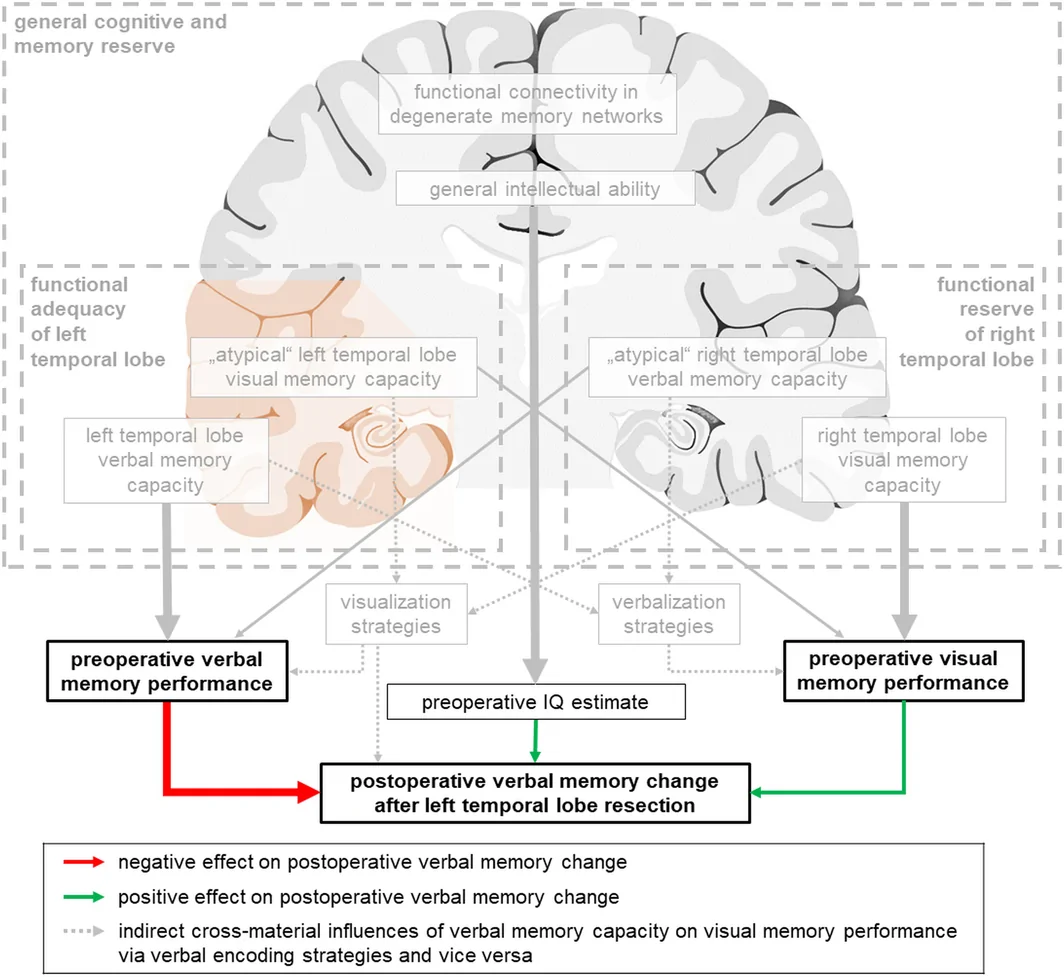
Conceptual summary figure of potential (i.e., hypothetical) mechanisms (presented in gray) behind predictors of postoperative verbal memory change after left temporal lobe resection due to mesial temporal sclerosis identified in the present study (presented in black). Thickness of arrows indicates the assumed relative amount of effect. IQ, intelligence quotient.

Previous studies have shown that educational level^7^ and IQ^5^, ^9^ are also associated with reduced postoperative verbal memory decline. Thus, the demonstrated protective effect of intact visual memory performance may not be specific to memory reserve alone but could reflect more general cognitive reserve. Unfortunately, our adult neuropsychological assessment did not include a full‐scale IQ measure but only an estimate of general intellectual ability (“IQ estimate”) derived from tests of logical reasoning and vocabulary. Exploratory analyses showed that inclusion of IQ estimate improved the prediction model compared to the model including only preoperative verbal and visual memory performance. However, the overall pattern of results regarding predictive value of preoperative verbal and visual memory performance did not change. This finding suggests that the influence of preoperative visual memory is largely independent of general cognitive reserve, which nevertheless seems to represent an additional protective factor against postoperative verbal memory decline. This shows that postoperative verbal memory change following left TLR is multifactorial and may depend not only on functional adequacy and reserve of the temporal lobes, but on a degenerate network spanning distributed regions of both hemispheres.^37^

In exploratory analyses, we replicated our findings in two‐factorial designs based on dichotomized preoperative verbal and/or visual memory impairments, corresponding to four different “memory phenotypes” with respect to material specificity: unimpaired memory, isolated verbal memory impairment, isolated visual memory impairment, and material‐unspecific (i.e., combined verbal and visual) memory impairment (see Figure 2B). Interestingly, less than half of our sample (42.86%) showed impaired verbal memory and only 20.00% exhibited a material‐specific verbal memory deficit (i.e., intact visual memory), which is often considered “typical” for left temporal lobe epilepsy according to the classical model of material specificity. Comparable proportions of verbal memory deficits have been reported previously.^39^ An explanation for this low proportion of verbal memory impairments in our sample could be that we only considered performance in delayed free recall. Indeed, one third of our cases with unimpaired performance in this measure showed impairments in other parameters of the same verbal memory test (i.e., learning, loss of words, or recognition). However, the low incidence of “typical” verbal memory deficits despite the presence of MTS also underscores that verbal memory is unlikely to be located exclusively in the left temporal lobe.

A recent study examined the influence of cognitive phenotypes spanning multiple cognitive domains on postoperative verbal memory change and found no benefit beyond prediction based on preoperative verbal memory alone.^10^ However, that study did not distinguish between material‐specific memory phenotypes. To our knowledge, the present study is the first to investigate the relationship between material‐specific memory phenotypes and postoperative verbal memory change. Our findings suggest that such a subdivision can be clinically relevant. In particular, we identified a subgroup of people with preoperative verbal memory impairments who nevertheless showed a substantial risk of postoperative verbal memory decline, namely those with additional visual memory impairments. In this subgroup, 33.33% experienced significant verbal memory decline, resulting in severe memory deficits due to the already low preoperative level. Thus, visual memory impairments may serve as “red flags” in people who would be considered at low risk for postoperative verbal memory decline when preoperative verbal memory alone is taken into account. Although such a category‐based approach may be easily applicable in clinical practice, it should be acknowledged that categorizing individuals into cognitive phenotypes based on predefined cutoffs of continuous test scores represents a considerable simplification of complex neuropsychological profiles. Therefore, careful evaluation at the individual level remains essential. This is also underlined by the overall low extent of variance explained by our models.

A few limitations should be considered when interpreting the generalizability of our findings. First, we examined a highly specific subgroup of cases with left‐sided anteromedial TLR due to MTS and typical (i.e., left‐hemispheric) language dominance. Consequently, the results may not generalize to other surgical approaches, to other etiologies, to right‐sided TLR, or to individuals with atypical language dominance. For example, resections sparing the hippocampus may have more favorable verbal memory outcomes,^5^, ^8^ individuals without MTS may experience more verbal memory decline,^13^ and right‐sided surgery can also affect postoperative verbal memory, albeit less frequently.^3^ Second, the findings may be influenced by the specific memory tests used in this study, although analyses based on dichotomized predictor and outcome variables are likely less susceptible to test‐specific effects. Furthermore, there are limitations associated with the retrospective data collection, including incomplete information on potentially confounding variables and the use of heterogeneous tests assessing cognitive domains other than memory, which could not be considered in our analyses.

## 5 CONCLUSIONS

Given the elective nature of epilepsy surgery, accurate prediction of individual risk for postoperative memory decline is essential for preoperative counseling of people with left temporal lobe epilepsy and typical language dominance. In addition to the well‐established material‐specific prediction of verbal memory outcome based on preoperative verbal memory performance, the present study demonstrates a cross‐material predictive value of preoperative visual memory performance for postoperative verbal memory change. High visual memory performance exerted a protective effect against verbal memory decline at both the group and individual levels. Furthermore, exploratory analyses indicated an additional protective influence of high general intellectual ability. The present findings highlight that from a clinical perspective, verbal memory scores should not be interpreted in isolation but in concert with visual memory and general, nonmemory cognitive functions. Specifically, the present findings demonstrate that poor preoperative verbal memory performance does not necessarily imply a low risk of postoperative verbal memory decline, which further depends on memory reserve and general cognitive reserve capacities. Therefore, even cases with preexisting verbal memory impairments should be considered at risk for postoperative verbal memory decline, if there are indicators of poor visual memory performance or general intellectual ability.

## AUTHOR CONTRIBUTIONS

**Lea Marie Reisch:** Conceptualization; methodology; writing—original draft preparation; writing—review & editing; formal analysis; data curation; visualization. **Anne Hagemann:** Formal analysis; methodology; writing—review & editing. **Johanna Lena Hopf:** Investigation; data curation; writing—review & editing. **Florian J. Muecke:** Investigation; data curation; writing—review & editing. **Friedrich G. Woermann:** Resources; writing—review & editing. **Christian G. Bien:** Resources; supervision; writing—review & editing. **Philip Grewe:** Conceptualization; writing—review & editing; investigation; data curation; methodology; supervision; project administration.

## CONFLICT OF INTEREST STATEMENT

None of the authors has any conflict of interest to disclose. We confirm that we have read the Journal's position on issues involved in ethical publication and affirm that this report is consistent with those guidelines.

## ETHICS APPROVAL

The study protocol was approved by the Ethics Committee of the Ärztekammer Westfalen‐Lippe, Münster (no. 024‐229‐f‐S), which granted a waiver of patient consent in accordance with North Rhine‐Westphalian law on health data protection.

## CONSENT

The requirement for patient consent was waived by the ethics committee of the Ärztekammer Westfalen‐Lippe, Münster (no. 024‐229‐f‐S), in accordance with North Rhine‐Westphalian law on health data protection.

## Supporting information




**TABLE S1.** Results of the backward elimination in the log‐linear analysis.
**TABLE S2.** Parameter estimates of the adopted model of the log‐linear analysis (Model 2).
**TABLE S3.** Results of hierarchical multiple linear regression to predict extent of postoperative verbal memory change including intelligence quotient estimate as additional predictor (*n* = 80).
**TABLE S4.** Results of logistic regression to predict significant postoperative verbal memory decline according to the reliable change index after inclusion of intelligence quotient estimate (*n* = 80).

## Data Availability

The data that support the findings of this study are available from the corresponding author upon reasonable request.

## References

[1] Wiebe S , Blume WT , Girvin JP , Eliasziw M . A randomized, controlled trial of surgery for temporal‐lobe epilepsy. N Engl J Med. 2001;345(5):311–318. 10.1056/NEJM200108023450501 11484687

[2] Téllez‐Zenteno JF , Dhar R , Wiebe S . Long‐term seizure outcomes following epilepsy surgery: a systematic review and meta‐analysis. Brain. 2005;128(5):1188–1198. 10.1093/brain/awh449 15758038

[3] Sherman EMS , Wiebe S , Fay‐McClymont TB , Tellez‐Zenteno J , Metcalfe A , Hernandez‐Ronquillo L , et al. Neuropsychological outcomes after epilepsy surgery: systematic review and pooled estimates. Epilepsia. 2011;52(5):857–869. 10.1111/j.1528-1167.2011.03022.x 21426331

[4] Hermann BP , Seidenberg M , Haltiner A , Wyler AR . Relationship of age at onset, chronologic age, and adequacy of preoperative performance to verbal memory change after anterior temporal lobectomy. Epilepsia. 1995;36(2):137–145. 10.1111/j.1528-1157.1995.tb00972.x 7821270

[5] Wagner K , Uherek M , Horstmann S , Kadish NE , Wisniewski I , Mayer H , et al. Memory outcome after hippocampus sparing resections in the temporal lobe. J Neurol Neurosurg Psychiatry. 2013;84(6):630–636. 10.1136/jnnp-2012-304052 23345282

[6] Ljunggren S , Andersson‐Roswall L , Imberg H , Samuelsson H , Malmgren K . Predicting verbal memory decline following temporal lobe resection for epilepsy. Acta Neurol Scand. 2019;140(5):312–319. 10.1111/ane.13146 31273754

[7] Busch RM , Hogue O , Miller M , Ferguson L , McAndrews MP , Hamberger M , et al. Nomograms to predict verbal memory decline after temporal lobe resection in adults with epilepsy. Neurology. 2021;97(3):e263–e274. 10.1212/WNL.0000000000012221 PMC8302146 34011574

[8] Helmstaedter C , Elger C , Hufnagel A , Zentner J , Schramm J . Different effects of left anterior temporal lobectomy, selective amygdalohippocampectomy, and temporal cortical lesionectomy on verbal learning, memory, and recognition. J Epilepsy. 1996;9(1):39–45.

[9] Davies KG , Bell BD , Bush AJ , Wyler AR . Prediction of verbal memory loss in individuals after anterior temporal lobectomy. Epilepsia. 1998;39(8):820–828. 10.1111/j.1528-1157.1998.tb01175.x 9701371

[10] Baxendale S , Thompson P . The association of cognitive phenotypes with postoperative outcomes after epilepsy surgery in patients with temporal lobe epilepsy. Epilepsy Behav. 2020;112:107386. 10.1016/j.yebeh.2020.107386 32911298

[11] Binder JR , Sabsevitz DS , Swanson SJ , Hammeke TA , Raghavan M , Mueller WM . Use of preoperative functional MRI to predict verbal memory decline after temporal lobe epilepsy surgery. Epilepsia. 2008;49(8):1377–1394. 10.1111/j.1528-1167.2008.01625.x PMC2943848 18435753

[12] Baxendale S , Thompson P , Harkness W , Duncan J . Predicting memory decline following epilepsy surgery: a multivariate approach. Epilepsia. 2006;47(11):1887–1894. 10.1111/j.1528-1167.2006.00810.x 17116029

[13] Elshorst N , Pohlmann‐Eden B , Horstmann S , Schulz R , Woermann F , McAndrews MP . Postoperative memory prediction in left temporal lobe epilepsy: the Wada test is of no added value to preoperative neuropsychological assessment and MRI. Epilepsy Behav. 2009;16(2):335–340. 10.1016/j.yebeh.2009.08.003 19751990

[14] Richardson MP , Strange BA , Thompson PJ , Baxendale SA , Duncan JS , Dolan RJ . Pre‐operative verbal memory fMRI predicts post‐operative memory decline after left temporal lobe resection. Brain. 2004;127(11):2419–2426. 10.1093/brain/awh293 15459025

[15] Bonelli SB , Powell RHW , Yogarajah M , Samson RS , Symms MR , Thompson PJ , et al. Imaging memory in temporal lobe epilepsy: predicting the effects of temporal lobe resection. Brain. 2010;133(4):1186–1199. 10.1093/brain/awq006 PMC2850579 20157009

[16] Stasenko A , Kaestner E , Arienzo D , Schadler AJ , Helm JL , Shih JJ , et al. Preoperative white matter network organization and memory decline after epilepsy surgery. J Neurosurg. 2023;139(6):1576–1587. 10.3171/2023.4.JNS23347 PMC10640663 37178024

[17] Baxendale S , Thompson P , Harkness W , Duncan J . The role of the intracarotid amobarbital procedure in predicting verbal memory decline after temporal lobe resection. Epilepsia. 2007;48(3):546–552. 10.1111/j.1528-1167.2006.00940.x 17346250

[18] Stroup E , Langfitt J , Berg M , McDermott M , Pilcher W , Como P . Predicting verbal memory decline following anterior temporal lobectomy (ATL). Neurology. 2003;60(8):1266–1273. 10.1212/01.wnl.0000058765.33878.0d 12707428

[19] Baxendale S . The impact of epilepsy surgery on cognition and behavior. Epilepsy Behav. 2008;12(4):592–599. 10.1016/j.yebeh.2007.12.015 18299253

[20] Saling MM . Verbal memory in mesial temporal lobe epilepsy: beyond material specificity. Brain. 2009;132(3):570–582. 10.1093/brain/awp012 19251757

[21] St‐Laurent M , McCormick C , Cohn M , Mišić B , Giannoylis I , McAndrews MP . Using multivariate data reduction to predict postsurgery memory decline in patients with mesial temporal lobe epilepsy. Epilepsy Behav. 2014;31:220–227. 10.1016/j.yebeh.2013.09.043 24210456

[22] Dupont S , Duron E , Samson S , Denos M , Volle E , Delmaire C , et al. Functional MR imaging or Wada test: which is the better predictor of individual postoperative memory outcome? Radiology. 2010;255(1):128–134. 10.1148/radiol.09091079 20308450

[23] Frings L , Wagner K , Halsband U , Schwarzwald R , Zentner J , Schulze‐Bonhage A . Lateralization of hippocampal activation differs between left and right temporal lobe epilepsy patients and correlates with postsurgical verbal learning decrement. Epilepsy Res. 2008;78(2):161–170. 10.1016/j.eplepsyres.2007.11.006 18180141

[24] Mechanic‐Hamilton D , Korczykowski M , Yushkevich PA , Lawler K , Pluta J , Glynn S , et al. Hippocampal volumetry and functional MRI of memory in temporal lobe epilepsy. Epilepsy Behav. 2009;16(1):128–138. 10.1016/j.yebeh.2009.07.012 PMC2749903 19674939

[25] Binder JR , Swanson SJ , Sabsevitz DS , Hammeke TA , Raghavan M , Mueller WM . A comparison of two fMRI methods for predicting verbal memory decline after left temporal lobectomy: language lateralization versus hippocampal activation asymmetry. Epilepsia. 2010;51(4):618–626. 10.1111/j.1528-1167.2009.02340.x PMC2945221 19817807

[26] Woermann FG , Jokeit H , Luerding R , Freitag H , Schulz R , Guertler S , et al. Language lateralization by Wada test and fMRI in 100 patients with epilepsy. Neurology. 2003;61(5):699–701. 10.1212/01.wnl.0000078815.03224.57 12963768

[27] Helmstaedter C , Lendt M , Lux S . VLMT: Verbaler Lern‐und Merkfähigkeitstest. Göttingen: Beltz Test GmbH; 2001.

[28] Schmidt M . Rey Auditory Verbal Learning Test: RAVLT: A Handbook: Western Psychological Services. 1996.

[29] Weidlich S , Derouiche A , Hartje W . DCS‐II, Diagnosticum für Cerebralschädigung–II, Ein figuraler visueller Lern‐und Gedächtnistest nach F. Hillers. Bern: Huber; 2011.

[30] Witt J‐A , Coras R , Schramm J , Becker AJ , Elger CE , Blümcke I , et al. The overall pathological status of the left hippocampus determines preoperative verbal memory performance in left mesial temporal lobe epilepsy. Hippocampus. 2014;24(4):446–454. 10.1002/hipo.22238 24375772

[31] Horn W . Leistungsprüfsystem: L‐P‐S. Göttingen: Hogrefe; 1983.

[32] Lehrl S . Mehrfachwahl‐Wortschatz‐Intelligenztest. MWT‐B. Erlangen: Straube; 1977.

[33] Petermann F . Wechsler Adult Intelligence Scale ‐ Fourth Edition. WAIS‐IV. Deutschprachige Adaptation der WAIS‐IV nach David Wechsler. Frankfurt a. M.: Pearson; 2012.

[34] Boucher O , Dagenais E , Bouthillier A , Nguyen DK , Rouleau I . Different effects of anterior temporal lobectomy and selective amygdalohippocampectomy on verbal memory performance of patients with epilepsy. Epilepsy Behav. 2015;52:230–235. 10.1016/j.yebeh.2015.09.012 26469799

[35] Chelune GJ . Hippocampal adequacy versus functional reserve: predicting memory functions following temporal lobectomy. Arch Clin Neuropsychol. 1995;10(5):413–432. 14588901

[36] Jokeit H , Ebner A , Holthausen H , Markowitsch HJ , Moch A , Pannek H , et al. Individual prediction of change in delayed recall of prose passages after left‐sided anterior temporal lobectomy. Neurology. 1997;49(2):481–487. 10.1212/wnl.49.2.481 9270581

[37] Audrain S , Barnett A , Mouseli P , McAndrews MP . Leveraging the resting brain to predict memory decline after temporal lobectomy. Epilepsia. 2023;64(11):3061–3072. 10.1111/epi.17767 37643922

[38] Jones MK . Imagery as a mnemonic aid after left temporal lobectomy: contrast between material‐specific and generalized memory disorders. Neuropsychologia. 1974;12(1):21–30. 10.1016/0028-3932(74)90023-2 4821186

[39] Castro LH , Silva LCAM , Adda CC , Banaskiwitz NHC , Xavier AB , Jorge CL , et al. Low prevalence but high specificity of material‐specific memory impairment in epilepsy associated with hippocampal sclerosis. Epilepsia. 2013;54(10):1735–1742. 10.1111/epi.12343 23980806

