# Supplementary material for: Cross‐material memory prediction: Influence of preoperative visual memory on postoperative verbal memory change after left anterior temporal lobe resection in people with epilepsy
Source: Epilepsia. 2026 Aug 10;67(11):6015–27. doi: 10.1002/epi.70426 (PMC13768891; doi:10.1002/epi.70426)
Supplement: Supplementary file 1 [file EPI-67-6015-s001.docx]

Description of normative samples of the verbal memory tests

The normative sample of the Verbal Learning and Memory Test (1) comprised 515 healthy people between the age of 6 and 79 years. Normative data are provided for age groups. The test manual reports that there were no differences in verbal memory performance between females and males in the normative sample, and only weak correlations with IQ, so these factors were not considered in the normative groups.

The normative sample of the *Diagnosticum für Cerebralschädigung* (2) comprised 871 healthy people between the age of 5 and 88 years. Normative data are provided for groups classified by age and education.

1.	Helmstaedter C, Lendt M, Lux S. VLMT: Verbaler Lern-und Merkfähigkeitstest. Göttingen: Beltz Test GmbH; 2001.

2.	Weidlich S, Derouiche A, Hartje W. DCS-II, Diagnosticum für Cerebralschädigung–II, Ein figuraler visueller Lern-und Gedächtnistest nach F. Hillers. Bern: Huber; 2011.

Table S1

*Results of the backwards elimination in the loglinear analysis*

| Step^a^ |  |  | Effects | *χ^2^* | df | *p* |
| --- | --- | --- | --- | --- | --- | --- |
| 0 | Generating Class^b^ |  | verbal memory impairment (pre)*visual memory impairment (pre)*verbal memory change (post) | 0.00 | 0 |  |
|  | Deleted Effect | 1 | verbal memory impairment (pre)*visual memory impairment (pre)*verbal memory change (post) | 2.31 | 1 | .128 |
| 1 | Generating Class^b^ |  | verbal memory impairment (pre)*visual memory impairment (pre),  verbal memory impairment (pre)*verbal memory change (post),  visual memory impairment (pre)*verbal memory change (post) | 2.31 | 1 | .128 |
|  | Deleted Effect | 1 | verbal memory impairment (pre)*visual memory impairment (pre) | 3.09 | 1 | .079 |
|  |  | 2 | verbal memory impairment (pre)*verbal memory change (post) | 8.20 | 1 | .004 |
|  |  | 3 | visual memory impairment (pre)*verbal memory change (post) | 5.44 | 1 | .020 |
| **2** | **Generating Class^b^** |  | **verbal memory impairment (pre)*verbal memory change (post),  visual memory impairment (pre)*verbal memory change (post)** | **5.40** | **2** | **.067** |
|  | Deleted Effect | 1 | verbal memory impairment (pre)*verbal memory change (post) | 6.52 | 1 | .011 |
|  |  | 2 | visual memory impairment (pre)*verbal memory change (post) | 3.76 | 1 | .052 |
| 3 | Generating Class^b^ |  | verbal memory impairment (pre)*verbal memory change (post),  visual memory impairment (pre) | 9.16 | 3 | .027 |
|  | Deleted Effect | 1 | verbal memory impairment (pre)*verbal memory change (post) | 6.52 | 1 | .011 |
|  |  | 2 | visual memory impairment (pre) | 0.47 | 1 | .494 |
| 4 | Generating Class^b^ |  | verbal memory impairment (pre)*verbal memory change (post) | 9.63 | 4 | .047 |
|  | Deleted Effect | 1 | verbal memory impairment (pre)*verbal memory change (post) | 6.52 | 1 | .011 |
| 5 | Generating Class^b^ |  | verbal memory impairment (pre)*verbal memory change (post) | 9.63 | 4 | .047 |

*Note.* a) At each step, the effect with the largest significance level for the Likelihood Ratio Change is deleted, provided the significance level is larger than .050. b) Statistics are displayed for the best model at each step after step 0. c). For 'Deleted Effect', this is the change in the *χ^2^* after the effect is deleted from the model. The adopted model (i.e., the last model fitting the data) is printed in bold.

Table S2

*Parameter estimates of the adopted model of the loglinear analysis (Model 2)*

| Parameter | Coeff. | SE | *Z*-Value | *p* | Lower 95 CI | Upper 95 CI |
| --- | --- | --- | --- | --- | --- | --- |
| verbal memory impairment (pre) * verbal memory change (post) | −0.28 | 0.11 | −2.46 | .014 | −0.50 | −0.06 |
| visual memory impairment (pre) * verbal memory change (post) | 0.20 | 0.11 | 1.92 | .055 | −0.00 | 0.41 |
| verbal memory impairment (pre) | 0.25 | 0.11 | 2.21 | .027 | 0.03 | 0.47 |
| visual memory impairment (pre) | 0.00 | 0.11 | 0.00 | .999 | −0.21 | 0.21 |
| verbal memory change (post) | 0.41 | 0.12 | 3.58 | < .001 | 0.19 | 0.64 |

Table S3

*Results of hierarchical multiple linear regression to predict extent of postoperative verbal memory change including IQ estimate as additional predictor (n = 80)*

|  | *B* | *SE B* | *β* | *p* | *R^2^* | *p* (*∆R^2^*) |
| --- | --- | --- | --- | --- | --- | --- |
| Block 1 |  |  |  |  | 0.21 | <.001 |
| (constant) | −2.64 | 0.34 |  | <.001 |  |  |
| Preoperative verbal memory | −0.95 | 0.21 | −.45 | <.001 |  |  |
| Block 2 |  |  |  |  | 0.25 | .027 |
| (constant) | −2.27 | 0.37 |  | <.001 |  |  |
| Preoperative verbal memory | −1.06 | 0.21 | −.50 | <.001 |  |  |
| Preoperative visual memory | 0.54 | 0.24 | .23 | .027 |  |  |
| Block 3 |  |  |  |  | 0.34 | .003 |
| (constant) | −2.48 | 0.36 |  | <.001 |  |  |
| Preoperative verbal memory | −1.14 | 0.20 | −.55 | <.001 |  |  |
| Preoperative visual memory | 0.50 | 0.23 | .21 | .031 |  |  |
| IQ estimate | 1.03 | 0.33 | .29 | .003 |  |  |
| Block 4 |  |  |  |  | 0.39 | .103 |
| (constant) | −1.04 | 0.85 |  | .224 |  |  |
| Preoperative verbal memory | −1.25 | 0.20 | −.60 | <.001 |  |  |
| Preoperative visual memory | 0.53 | 0.23 | .22 | .024 |  |  |
| IQ estimate | 1.17 | 0.34 | .33 | .001 |  |  |
| Age at surgery | −0.04 | 0.02 | −.17 | .114 |  |  |
| Age at epilepsy onset | −0.03 | 0.02 | −.12 | .253 |  |  |
| Preoperative focal to bilateral tonic-clonic seizures | 0.50 | 0.55 | .09 | .369 |  |  |

*Note*. *B*: unstandardized coefficient, *SE*: standard error, *β*: standardized coefficient, *R^2^*: determination coefficient, ∆*R^2^*: change in *R^2^*. All variance inflation factors < 1.36.

Table S4

*Results of logistic regression to predict significant postoperative verbal memory decline according to the RCI after inclusion of IQ estimate (n = 80)*

|  | *B* | *SE B* | Odds ratio | *p* | *χ^2^* | *p* (*∆χ2*) |
| --- | --- | --- | --- | --- | --- | --- |
| Block 1 |  |  |  |  | 8.48 | .004 |
| (constant) | −0.18 | 0.28 |  | .525 |  |  |
| Preoperative verbal memory | 0.57 | 0.21 | 1.77 | .008 |  |  |
| Block 2 |  |  |  |  | 11.24 | .097 |
| (constant) | −0.44 | 0.33 |  | .183 |  |  |
| Preoperative verbal memory | 0.64 | 0.22 | 1.91 | .003 |  |  |
| Preoperative visual memory | −0.36 | 0.22 | 0.70 | .107 |  |  |
| Block 3 |  |  |  |  | 20.65 | .002 |
| (constant) | −0.28 | 0.35 |  | .416 |  |  |
| Preoperative verbal memory | 0.86 | 0.26 | 2.35 | .001 |  |  |
| Preoperative visual memory | −0.35 | 0.23 | 0.70 | .124 |  |  |
| IQ estimate | −1.14 | 0.41 | 0.32 | .005 |  |  |
| Block 4 |  |  |  |  | 24.76 | .250 |
| (constant) | −1.74 | 0.97 |  | .074 |  |  |
| Preoperative verbal memory | 0.98 | 0.28 | 2.65 | <.001 |  |  |
| Preoperative visual memory | −0.35 | 0.24 | 0.70 | .151 |  |  |
| IQ estimate | −1.38 | 0.47 | 0.25 | .003 |  |  |
| Age at surgery | 0.03 | 0.03 | 1.03 | .223 |  |  |
| Age at epilepsy onset | 0.02 | 0.02 | 1.02 | .335 |  |  |
| Preoperative focal to bilateral tonic-clonic seizures | −0.05 | 0.62 | 0.95 | .934 |  |  |

*Note*. *B*: unstandardized coefficient, *SE*: standard error, *χ^2^*: Model *χ^2^*, ∆*χ^2^*: change in *χ^2^*.
